# Weight change and fracture risk in patients with diabetic kidney disease: A nationwide population-based study

**DOI:** 10.3389/fmed.2022.912152

**Published:** 2022-07-28

**Authors:** Chang Seong Kim, Hong Sang Choi, Eun Hui Bae, Seong Kwon Ma, Bongseong Kim, Kyung-Do Han, Soo Wan Kim

**Affiliations:** ^1^Department of Internal Medicine, Chonnam National University Medical School, Gwangju, South Korea; ^2^Department of Internal Medicine, Chonnam National University Hospital, Gwangju, South Korea; ^3^Department of Statistics and Actuarial Science, Soongsil University, Seoul, South Korea

**Keywords:** diabetes, fracture, kidney disease, national health programs, weight

## Abstract

**Background:**

The increased risk of fracture has been associated with weight loss in patients with diabetes or chronic kidney disease. However, the relationship between weight changes over time and fracture risk in patients with diabetic kidney disease is still unknown.

**Methods:**

A total number of 78,922 patients with diabetic kidney disease, aged ≥ 40 years, were selected using the Korean National Health Insurance Service database, between 2009 and 2012. They were followed up until the end of 2018. Weight change was defined as the difference in body weight from the index year to 2 years later. Weight changes were then divided into five categories, ranging from weight loss of ≥10% to weight gain of ≥10%.

**Results:**

Fractures were identified in 9,847 patients with diabetic kidney disease, over a median follow-up of 5.2 years. The risk of composite fracture of the vertebral, hip, or other sites increased as the weight change increased. Specifically, patients with ≥10% weight loss (hazard ratio [HR], 1.286; 95% confidence interval [CI], 1.184–1.398) and ≥10% weight gain (HR, 1.198; 95% CI, 1.080–1.330) showed a higher HR compared to those with ≤ 5% weight change after adjusting for several confounding factors. Higher HR of vertebral and hip fractures was also seen with increased weight loss or gain. In particular, patients with ≥10% weight loss showed the highest HR for hip fractures (HR, 1.738; 95% CI, 1.489–2.028).

**Conclusions:**

Both weight loss and weight gain increase the risk of fracture in patients with diabetic kidney disease. Therefore, patients with diabetic kidney disease who experience weight changes should be made aware of the risk of fracture.

## Introduction

The increasing prevalence of diabetic kidney disease (DKD) parallels the dramatic worldwide rise in the occurrence of diabetes (1, 2). The global leading cause of chronic kidney disease (CKD) is DKD and it is known to develop in approximately 40% of patients with diabetes (1, 3). In later stages, complications and natural history of DKD is different from those of other non-diabetic CKD. For example, anemia often develops earlier in DKD compared to other types of CKD due to the predominant tubulointerstitial nature of DKD (4). Moreover, deaths from cardiovascular disease and infections are highly prevalent in those patients (1, 4).

Bone fractures are concerning events that have devastating health consequences. Although factors such as age, sex, comorbidities, and poor health status play a role, it is the fracture event itself that is responsible for an increased mortality risk (5, 6). Declining kidney function is associated with abnormalities in bone and mineral metabolism that predisposes patients to a greater risk of fractures, particularly hip fractures (7–9). Furthermore, adynamic bone disease accompanies DKD, which can cause lower parathyroid hormone levels because advanced glycation end products inhibit parathyroid hormone secretion (10), and thereby result in an increased risk of fragility fractures (11). Diabetes is also associated with sarcopenia, hypoglycemia, retinopathy, and peripheral neuropathy, which may increase the risk of fractures caused by falls (12, 13).

In addition to the traditional fracture risk factors, such as age, body mass index (BMI), comorbid conditions, fracture and fall history, and bone mineral density (BMD), a recent study showed that weight change is associated with increased fracture incidence in postmenopausal women (14). Moreover, previous studies have shown that increased fibroblast growth factor-23, sclerostin, activin A, and altered circadian rhythm are potential risk factors for fractures in DKD patients (11). However, the association of weight change with fracture risk has not been well-studied in this population.

Our objective was therefore to investigate the association between loss or gain of body weight (BW) over 2 years and the subsequent incidence of overall fracture. Furthermore, we aimed to determine whether weight change would increase the incidence of specific fractures classified by vertebral, hip, and other sites in DKD patients selected from a nationwide cohort.

## Methods

### Korean National Health Insurance Service data

We used the national health insurance claims database established by the Korean National Health Insurance Service (NHIS), in our study. This database includes data on all claims provided by the NHIS and Medical Aid programs. The Korean NHIS database represents the entire South Korean population and the details of this database have been previously described (15, 16). Korean citizens who are insured, undergo health examinations that are supported by the NHIS annually or biennially, depending on their occupation.

### Study participants

**Figure 1:**
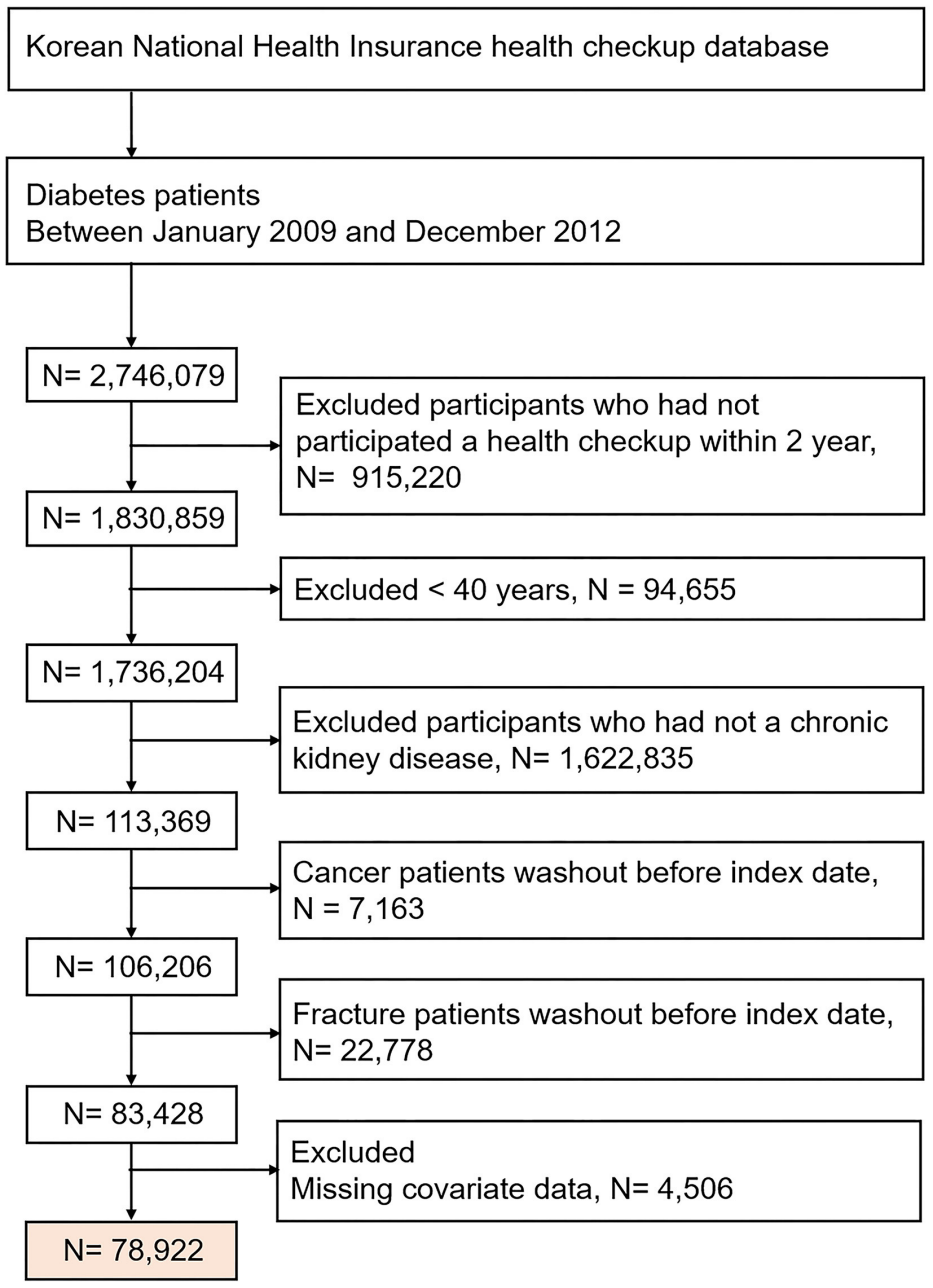
Flowchart of participant enrolment.

We initially identified 2,746,079 patients with diabetes, who between 2009 and 2012 underwent a health check-up. Of these, we excluded the following patients from our study: patients who did not undergo a repeat health check-up after 2 years, those without a history of CKD, those with malignancy, those with a history of fracture before the index date, patients < 40 years of age (fractures are rare in this subgroup), and patients who had any health examination data missing. From these vast data results, we finally included 78,922 patients with DKD in our study. The date of the last health check-up was set as the index date. The selection of study participants is presented in a detailed flowchart in Figure 1. The participant follow-up continued and was only concluded when one of the following occurred: fracture, death, loss of health insurance qualification, or end of study (December 31, 2018).

### Definitions

Weight change was calculated over a 2-year interval based on the weight difference between the first and the second health check-up (expressed as a percentage). Based on a previous study (17), patients with <5% weight change were defined as having a stable weight and all participants were divided into the following five groups: ≥10% weight loss, 5–10% weight loss, <5% weight change, 5–10% weight gain, and ≥10% weight gain. The amount of intentional weight loss or gain among participants in this study was unknown.

Hypertension was defined as a previous diagnosis of hypertension according to the International Classification of Diseases, 10th Revision, Clinical Modification (ICD-10-CM) codes (I10–I13, I15) and a history of antihypertensive drug use, or a recorded systolic blood pressure (BP) ≥ 140 mmHg, or diastolic BP ≥ 90 mmHg. Diabetes mellitus was defined as a previous clinical diagnosis (ICD-10-CM codes E11–14) and a treatment history of diabetes or a recorded fasting serum glucose level ≥ 126 mg/dL. CKD was calculated using the Modification of Diet in Renal Disease formula and was defined as an estimated glomerular filtration rate (eGFR) <60 mL/min/1.73 m^2^ (18). Dyslipidemia was defined as follows: the presence of ICD-10-CM code E78, a history of lipid-lowering drug use, or a total serum cholesterol concentration of ≥240 mg/dL. The lowest 20% of the socio-economic status was defined as low-income. End-stage renal disease (ESRD) was defined as the presence of hemodialysis, peritoneal dialysis, or kidney transplantation. Patients with ESRD were identified using a combination of ICD-10-CM codes (N18–19, Z49, Z94.0, and Z99.2) and a special code (V001, procedure-related outpatient care or inpatient treatment on the day of hemodialysis; V003, peritoneal dialysis; and V005, kidney transplantation). BMI was calculated as weight (in kg) divided by the square of the height (in m). Smoking history was categorized as follows: never, former, or current smoker. Alcohol consumption was categorized into none, moderate, or heavy drinkers (≥30 g of alcohol/day). At least 20 min of physical activity per day, until almost out of breath, and more than 3 days of physical activity during the previous week, was considered regular exercise.

### Outcomes

The incidence of fracture was the study endpoint. Vertebral fracture was defined as more than two claims per year of the following ICD-10-CM codes: S22.0, S32.0, S32.1, M48.4, and M48.5, while hip fracture was defined as claims of the following diagnostic codes: S72.0, S72.1, and S72.2 at admission. -Other fractures were defined as more than two claims per year of the following ICD-10-CM codes: S42.0, S42.2, S42.3, S52.5, S52.6, S82.3, S82.5, and S82.6. Lastly, composite fracture of the vertebral, hip, or other sites was defined as any-fracture. We excluded traumatic fractures in this study.

### Statistical analyses

Data are presented as mean ± standard deviation for continuous variables and numbers with proportions for categorical variables. Non-normally distributed variables are presented as geometric means (95% confidence interval [CI]). Chi-square test or analysis of variance was used to test inter-group differences, as appropriate. The incidence rate of fractures is presented per 1,000 person-years. Multivariable Cox proportional hazard regression analysis was used to estimate the hazard ratios (HRs) and 95% CIs of weight change associated fracture risk after adjusting for age, sex, smoking, alcohol consumption, regular exercise, low-income status, use of insulin, number of oral hypoglycemic agents, duration of diabetes, previous history of hypertension, dyslipidemia and ESRD, and previous BW. Sensitivity analyses were performed after excluding patients who had fractures within 1-year of follow-up. Furthermore, we also performed analyses to identify whether there was an association between the risk of fractures and weight change over 4-years between health check-ups. Subgroup analyses were performed to assess the effect modification on the risk of fractures in DKD patients based on age (<65 and ≥65-years groups), sex, history of hypertension, duration of diabetes (<5 and ≥5-year groups), the presence of proteinuria, BMI (<25 and ≥25 kg/m^2^), the use of insulin, and the number of oral hypoglycemic agents (≥3) used. Interaction terms were added to test for effect modification across subgroups (P for interaction <0.05 was considered statistically significant). All data analyses were conducted using the SAS software (version 9.4; SAS Institute, Cary, NC), and P < 0.05 was considered statistically significant.

## Results

### Baseline characteristics according to the status of weight changes

Table 1 presents the demographic, behavioral, and health examination characteristics of the participants at the index year according to weight change. The mean age of all patients was 68.1 ± 9.0 years, 52.3% of them were males, and the mean eGFR of all patients was 45.3 ± 14.2 mL/min/1.73 m^2^ and the mean BMI was 25.0 ± 3.3 kg/m^2^. In our population, the percentage of patients with < 5% weight change over the 2-year period was 69.5%, while ≥ 10% weight loss was 4.7%, 5–10% weight loss was 14.0%, 5–10% weight gain was 8.7%, and ≥10% weight gain was 3.1%. The stable weight group participants, according to our results, tended to be younger, smokers, alcohol consumers, participated in regular exercise, had a low-income, and had a higher baseline eGFR compared with participants in other weight loss or weight gain groups. However, the following increased with weight gain: the prevalence of hypertension and dyslipidemia, BMI, waist circumference, and total cholesterol and triglyceride levels.

**Table 1:** Baseline characteristics of the study population according to the status of weight changes.

**Characteristics**	**Weight change**						
	**Total**	≥**-10%**	−**10 to** −**5%**	−**5 to 5%**	**5 to 10%**	≥**10%**	***P*** **-value**
	**(*****n*** = **78,922)**	**(*****n*** = **3,683)**	**(*****n*** = **11,055)**	**(*****n*** = **54,887)**	**(*****n*** = **6,833)**	**(*****n*** = **2,464)**	
Age, years	68.06 ± 8.99	70.85 ± 9.29	69.2 ± 8.82	67.65 ± 8.93	67.78 ± 9.04	68.49 ± 9.44	<0.001
Sex, male	41,262 (52.28)	1,405 (38.15)	5,173 (46.79)	30,186 (55.0)	3,405 (49.83)	1,093 (44.36)	<0.001
Smoking							
Never	52,448 (66.46)	2,810 (76.3)	7,767 (70.26)	35,474 (64.63)	4,640 (67.91)	1,757 (71.31)	
Former	16,844 (21.34)	507 (13.77)	2,003 (18.12)	12,454 (22.69)	1,432 (20.96)	448 (18.18)	<0.001
Current	9,630 (12.20)	366 (9.94)	1,285 (11.62)	6,959 (12.68)	761 (11.14)	259 (10.51)	
Alcohol consumption							
None	60,912 (77.18)	3,236 (87.86)	9,049 (81.85)	41,167 (75.00)	5,382 (78.76)	2,078 (84.33)	
Moderate	15,522 (19.67)	399 (10.83)	1,745 (15.78)	11,791 (21.48)	1,256 (18.38)	331 (13.43)	<0.001
Heavy	2,488 (3.15)	48 (1.3)	261 (2.36)	1,929 (3.51)	195 (2.85)	55 (2.23)	
Regular exercise	16,981 (21.52)	558 (15.15)	2,171 (19.64)	12,549 (22.86)	1,304 (19.08)	399 (16.19)	<0.001
Hypertension	67,272 (85.24)	3,160 (85.8)	9,381 (84.86)	46,669 (85.03)	5,894 (86.26)	2,168 (87.99)	<0.001
Dyslipidemia	47,841 (60.62)	2,119 (57.53)	6,543 (59.19)	33,297 (60.66)	4,291 (62.8)	1,591 (64.57)	<0.001
ESRD	3,133 (3.97)	252 (6.84)	516 (4.67)	1,867 (3.4)	325 (4.76)	173 (7.02)	<0.001
Low income	13,839 (17.54)	699 (18.98)	1,976 (17.87)	9,379 (17.09)	1,295 (18.95)	490 (19.89)	<0.001
eGFR, ml/min 1.73m^2^	45.29 ± 14.17	43.34 ± 14.68	45.17 ± 13.94	45.67 ± 14.15	44.43 ± 13.97	42.75 ± 14.67	<0.001
BMI, kg/m^2^	25.04 ± 3.26	22.48 ± 3.26	24.01 ± 3.10	25.25 ± 3.11	25.9 ± 3.34	26.39 ± 3.87	<0.001
WC, cm	86.33 ± 8.62	82.02 ± 9.38	84.12 ± 8.52	86.74 ± 8.36	88.02 ± 8.67	88.71 ± 9.65	<0.001
SBP, mmHg	130.85 ± 16.56	128.62 ± 17.54	129.60 ± 16.58	130.97 ± 16.37	132.37 ± 16.76	132.83 ± 17.71	<0.001
DBP, mmHg	76.94 ± 10.35	75.76 ± 10.8	76.30 ± 10.45	77.04 ± 10.25	77.53 ± 10.49	77.76 ± 10.81	<0.001
Fasting glucose, mg/dL	131.2 ± 47.92	131.18 ± 58.84	129.96 ± 51.87	131.44 ± 46.18	130.93 ± 46.90	132.27 ± 51.89	0.0375
Total cholesterol, mg/dL	179.87 ± 43.69	177.30 ± 42.93	178.51 ± 42.80	180.13 ± 43.27	180.82 ± 48.61	181.20 ± 43.55	<0.001
Triglyceride, mg/dL	141.19 (140.69–141.69)	128.25 (126.24–130.29)	132.52 (131.26– 133.79)	143.1 (142.49– 143.71)	145.92 (144.15– 147.71)	146.57 (143.72– 149.49)	<0.001
HDL, mg/dL	47.19 ± 13.75	48.18 ± 16.37	47.9 ± 14.08	47.03 ± 13.3	46.86 ± 13.59	47.05 ± 17.64	<0.001
Low-density lipoprotein, mg/dL	100.95 ± 43.27	100.81 ± 53.63	100.44 ± 36.96	100.98 ± 42.57	101.47 ± 51.15	101.52 ± 43.62	0.556
Insulin use	20,694 (26.22)	1,230 (33.4)	2,993 (27.07)	13,172 (24)	2,304 (33.72)	995 (40.38)	<0.001
Anti-diabetes medication							
Sulfonylurea	49,703 (62.98)	2,355 (63.94)	6,968 (63.03)	34,430 (62.73)	4,326 (63.31)	1,624 (65.91)	0.015
Metformin	53,535 (67.83)	2,665 (72.36)	7,729 (69.91)	36,981 (67.38)	4,531 (66.31)	1,629 (66.11)	<0.001
Meglitinides	4,555 (5.77)	279 (7.58)	626 (5.66)	2,914 (5.31)	506 (7.41)	230 (9.33)	<0.001
Thiazolidinedione	7,917 (10.03)	373 (10.13)	1,039 (9.4)	5,074 (9.24)	1,034 (15.13)	397 (16.11)	<0.001
Dipeptidyl Peptidase-4	19,518 (24.73)	1,011 (27.45)	2,937 (26.57)	13,180 (24.01)	1,786 (26.14)	604 (24.51)	<0.001
α-glucosidase inhibitors	18,002 (22.81)	955 (25.93)	2,534 (22.92)	12,121 (22.08)	1,703 (24.92)	689 (27.96)	<0.001
Number of OHA ≥ 3	26,300 (33.32)	1,418 (38.5)	3,826 (34.61)	17,592 (32.05)	2,501 (36.6)	963 (39.08)	<0.001

ESRD, end-stage renal disease; eGFR; estimated glomerular filtration rate; BMI, body mass index; WC, waist circumference; SBP, systolic blood pressure; DBP, diastolic blood pressure; HDL, High-density lipoprotein; OHA, oral hypoglycemic agents.

### Weight changes and risk of any, vertebral, hip, or other site fractures

The median follow-up duration was 5.2 years, and we identified any-fracture in 9,847 participants over the period of observation. The association between weight change and the incidence and risk of fracture in DKD patients is presented in Table 2. When participants with <5% weight change for 2-years were used as the reference group, the incidence of any-fracture increased with both weight-loss and weight-gain. This association was maintained when specific fracture sites, vertebral, hip, or other sites were considered.

**Table 2:** Incidence rates and hazard ratios of fractures according to the status of weight changes.

**Weight change**	**Number**	**Fracture**	**Follow-up duration, person-years**	**Incidence rate, per 1,000 person-years**	**Model 1, HR (95% CI)** ^b^	**Model 2, HR (95% CI)** ^c^	**Model 3, HR (95% CI)** ^d^
Any-fracture^a^							
≥-10%	3,683	619	17294.30	35.79	1.341 (1.234, 1.457)	1.305 (1.201, 1.418)	1.286 (1.184, 1.398)
−10 to −5%	11,055	1,546	57463.52	26.90	1.117 (1.056, 1.181)	1.106 (1.046, 1.170)	1.100 (1.040, 1.163)
−5 to 5%	54,887	6,415	297739.06	21.55	1 (Reference)	1 (Reference)	1 (Reference)
5 to 10%	6,833	889	35875.71	24.78	1.103 (1.028, 1.183)	1.063 (0.991, 1.140)	1.060 (0.988, 1.137)
≥10%	2,464	378	12331.42	30.65	1.279 (1.153, 1.420)	1.211 (1.091, 1.344)	1.198 (1.080, 1.330)
Vertebral facture							
≥-10%	3,683	214	18354.13	11.66	1.340 (1.163, 1.544)	1.308 (1.135, 1.508)	1.303 (1.130, 1.502)
−10 to −5%	11,055	514	60470.73	8.50	1.124 (1.020, 1.239)	1.114 (1.011, 1.228)	1.112 (1.009, 1.226)
−5 to 5%	54,887	2,024	310637.98	6.52	1 (Reference)	1 (Reference)	1 (Reference)
5 to 10%	6,833	281	37677.38	7.46	1.086 (0.958, 1.230)	1.059 (0.935, 1.200)	1.058 (0.934, 1.199)
≥10%	2,464	123	13015.67	9.45	1.255 (1.046, 1.506)	1.219 (1.016, 1.463)	1.215 (1.012, 1.459)
Hip fracture							
≥-10%	3,683	191	18551.44	10.30	1.890 (1.621, 2.204)	1.794 (1.538, 2.093)	1.738 (1.489, 2.028)
−10 to −5%	11,055	362	61157.24	5.92	1.269 (1.128, 1.427)	1.242 (1.104, 1.397)	1.225 (1.089, 1.378)
−5 to 5%	54,887	1,236	313677.89	3.94	1 (Reference)	1 (Reference)	1 (Reference)
5 to 10%	6,833	171	38149.50	4.48	1.087 (0.926, 1.276)	1.018 (0.868, 1.195)	1.012 (0.862, 1.188)
≥10%	2,464	93	13146.42	7.07	1.550 (1.255, 1.914)	1.4 (1.133, 1.730)	1.360 (1.101, 1.681)
Other fractures							
≥-10%	3,683	295	18157.34	16.25	1.130 (1.003, 1.273)	1.105 (0.981, 1.245)	1.092 (0.969, 1.230)
−10 to −5%	11,055	849	59334.05	14.31	1.071 (0.994, 1.154)	1.066 (0.989, 1.149)	1.061 (0.984, 1.143)
−5 to 5%	54,887	3,765	304881.86	12.35	1 (Reference)	1 (Reference)	1 (Reference)
5 to 10%	6,833	522	36848.21	14.17	1.103 (1.006, 1.208)	1.063 (0.970, 1.166)	1.061 (0.968, 1.163)
≥10%	2,464	201	12772.02	15.74	1.171 (1.016, 1.350)	1.111 (0.963, 1.281)	1.101 (0.955, 1.270)

a Composite fracture of the vertebral, hip, or other sites was defined as any-fracture.

b Model 1, adjusted for age and sex.

c Model 2, adjusted for age, sex, smoking, alcohol consumption, regular exercise, low-income status, use of insulin, number of oral hypoglycemic agents, diabetes duration, and previous histories of hypertension and dyslipidemia.

d Model 3, adjusted for age, sex, smoking, alcohol consumption, regular exercise, low-income status, use of insulin, number of oral hypoglycemic agents, duration of diabetes, previous histories of hypertension, dyslipidemia and end-stage renal disease, and previous body weight.

CI, confidential interval; HR, hazard ratio.

After adjusting for the participant's age, sex, health behavior factors, comorbidities including ESRD, duration of diabetes, anti-diabetic medications, and baseline BW (Cox model 3), the adjusted HRs for any-fracture increased to 1.100 (95% CI, 1.040–1.163) and 1.286 (95% CI, 1.184–1.398) in participants with 5–10% weight loss and ≥10% weight loss, respectively. Similarly, participants with ≥10% weight gain demonstrated a significantly increased risk of any-fracture compared to participants with stable weight during the period of observation (adjusted HRs [95% CIs]: 1.198 [1.080–1.330]).

Subsequent evaluation of the risk of vertebral, hip, or other site fractures according to weight changes using the multivariable Cox analysis revealed that participants with ≥10% weight loss had the highest risk of vertebral and hip fractures relative to the reference group (adjusted HRs [95% CIs]: 1.303 [1.130–1.502] and 1.738 [1.489–2.028], respectively), similar to the any-fracture risk. Moreover, participants with ≥10% weight gain also showed an increased risk of vertebral and hip fractures. However, weight changes did not significantly correlate with the risk of fracture at any other sites.

### Sensitivity analyses

To account for the possibility of reverse causation, a sensitivity analysis was performed, and participants who had fractures within 1-year of follow-up were excluded (Table 3). Even after the full adjustment, compared to the stable weight group, the ≥10% weight loss and ≥10% weight gain groups had a higher HRs for any, vertebral, or hip fracture in DKD patients. Additionally, these analyses were repeated to confirm the association between weight change over 4-years and fracture risk (Supplementary Table 1). The adjusted HRs for any-fracture were 1.253 (95% CI, 1.134–1.384) and 1.081 (95% CI, 1.002–1.166) in participants with ≥10% weight loss and 5–10% weight loss, respectively. This association was retained when vertebral and hip fracture risk was considered. However, fracture risk only tended to increase in participants with ≥10% weight gain, but was not significant for any-fracture or hip fracture after full adjustment in the sensitivity analyses.

**Table 3:** Sensitivity analysis of incidence rates and hazard ratios of fractures according to the status of weight changes after excluding fractures within 1 year of follow-up.

**Weight change**	**Number**	**Fracture**	**Follow-up duration, person-years**	**Incidence rate, per 1,000 person-years**	**Model 1, HR (95% CI)** ^b^	**Model 2, HR (95% CI)** ^c^	**Model 3, HR (95% CI)** ^d^
Any-fracture^a^							
≥-10%	3,395	513	13750.85	37.31	1.360 (1.242, 1.490)	1.324 (1.209, 1.451)	1.307 (1.193, 1.433)
−10 to −5%	10,561	1,268	46645.89	27.18	1.098 (1.032, 1.167)	1.088 (1.023, 1.157)	1.082 (1.018, 1.151)
−5 to 5%	53,228	5,383	243634.63	22.09	1 (Reference)	1 (Reference)	1 (Reference)
5 to 10%	6,572	721	29167.24	24.72	1.072 (0.992, 1.158)	1.033 (0.955, 1.116)	1.030 (0.953, 1.114)
≥10%	2,342	322	9933.39	32.42	1.318 (1.177, 1.475)	1.246 (1.113, 1.395)	1.234 (1.102, 1.382)
Vertebral facture							
≥-10%	3,395	172	14554.73	11.82	1.317 (1.125, 1.542)	1.285 (1.097, 1.506)	1.280 (1.093, 1.500)
−10 to −5%	10,561	421	48738.81	8.64	1.110 (0.998, 1.236)	1.100 (0.988, 1.225)	1.099 (0.987, 1.223)
−5 to 5%	53,228	1,690	252828.51	6.68	1 (Reference)	1 (Reference)	1 (Reference)
5 to 10%	6,572	227	30365.53	7.48	1.061 (0.924, 1.219)	1.035 (0.901, 1.189)	1.034 (0.900, 1.189)
≥10%	2,342	105	10458.71	10.04	1.298 (1.066, 1.581)	1.26 (1.034, 1.536)	1.256 (1.031, 1.531)
Hip fracture							
≥-10%	3,395	157	14668.15	10.70	1.857 (1.568, 2.199)	1.778 (1.501, 2.107)	1.731 (1.461, 2.052)
−10 to −5%	10,561	299	49218.47	6.08	1.236 (1.087, 1.406)	1.216 (1.069, 1.383)	1.202 (1.056, 1.367)
−5 to 5%	53,228	1,054	254942.27	4.13	1 (Reference)	1 (Reference)	1 (Reference)
5 to 10%	6,572	148	30679.65	4.82	1.112 (0.936, 1.321)	1.046 (0.88, 1.243)	1.041 (0.876, 1.237)
≥10%	2,342	81	10546.14	7.68	1.594 (1.271, 1.999)	1.453 (1.158, 1.823)	1.421 (1.132, 1.783)
Other fractures							
≥-10%	3,395	247	14372.49	17.19	1.193 (1.047, 1.358)	1.165 (1.022, 1.327)	1.153 (1.012, 1.314)
−10 to −5%	10,561	678	48032.58	14.12	1.053 (0.968, 1.144)	1.047 (0.963, 1.138)	1.042 (0.959, 1.133)
−5 to 5%	53,228	3,086	248931.93	12.40	1 (Reference)	1 (Reference)	1 (Reference)
5 to 10%	6,572	413	29854.75	13.83	1.072 (0.967, 1.188)	1.032 (0.931, 1.144)	1.030 (0.929, 1.142)
≥10%	2,342	165	10293.28	16.03	1.189 (1.016, 1.390)	1.123 (0.959, 1.314)	1.115 (0.953, 1.305)

a Composite fracture of the vertebral, hip, or other sites was defined as any-fracture.

b Model 1, adjusted for age and sex.

c Model 2, adjusted for age, sex, smoking, alcohol consumption, regular exercise, income status, use of insulin, more than three classes of oral hypoglycemic agents, diabetes duration, and previous histories of hypertension and dyslipidemia.

d Model 3, adjusted for age, sex, smoking, alcohol consumption, regular exercise, income status, use of insulin, more than three classes of oral hypoglycemic agents, diabetes duration, previous histories of hypertension, dyslipidemia and end-stage renal disease, and previous body weight.

CI, confidential interval; HR, hazard ratio.

### Subgroup analyses

**Figure 2:**
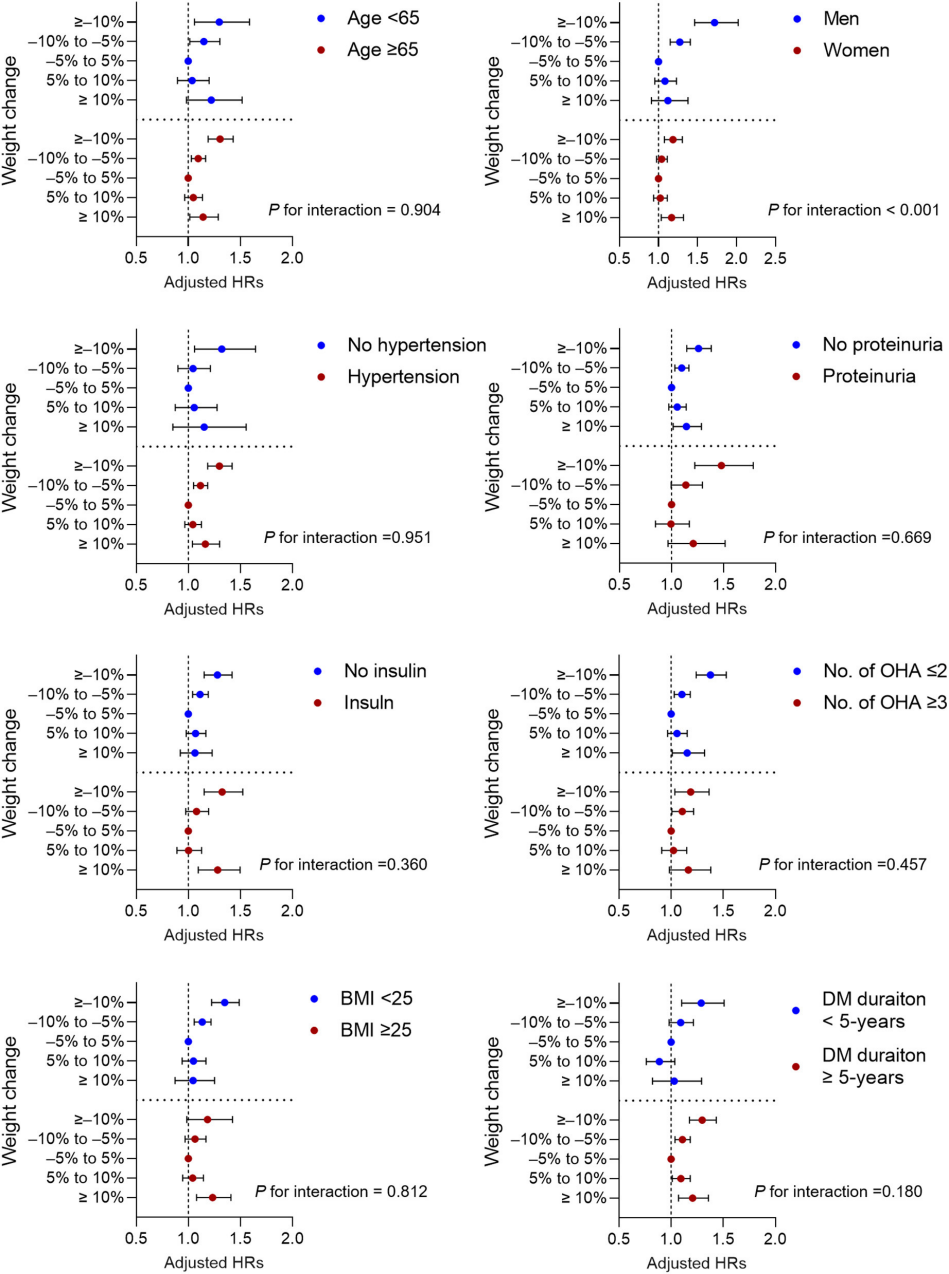
Subgroup analysis for adjusted hazard ratios (HRs) of incidence any fracture. Horizontal lines represent the range for 95% confidence intervals. Models were adjusted for age, sex, smoking, alcohol consumption, regular exercise, income status, use of insulin, more than three classes of oral hypoglycemic agents, diabetes duration, previous histories of hypertension, dyslipidemia and end-stage renal disease, and previous body weight. OHA, oral hypoglycemic agents; BMI, body mass index; DM, diabetes mellitus.

The association between weight change and the risk of any-fracture was explored, using subgroup analyses, after stratification by age, BMI, sex, diabetes duration, presence of proteinuria, history of hypertension, number of oral hypoglycemic agents, and insulin use (Figure 2). In all subgroup analyses, a high weight loss or high weight gain was consistently associated with the risk of any-fracture. The correlation between weight change and any-fracture risk was significantly stronger in men (P for interaction < 0.001). Higher weight loss, but not weight gain, was associated with increased any-fracture risk in patients without obesity (BMI < 25 kg/m^2^). In contrast, in patients with obesity (BMI ≥ 25 kg/m^2^), the ≥10% weight gain group had a higher HR, but the weight loss group had no significant association with any-fracture risk.

## Discussion

In this nationwide population-based cohort study, we found that DKD patients with ≥10% weight loss over 2-years had a 29% higher risk of any-fracture, 30% higher risk of vertebral fracture, and 74% higher risk of hip fracture compared to patients with < 5% weight change. Similarly, DKD patients with ≥10% weight gain had a 20% higher risk of any-fracture, 22% higher risk of vertebral fracture, and 36% higher risk of hip fracture compared to patients those who had a stable BW. Moreover, compared to the stable weight group, ≥10% weight gain was associated with an increased risk of any-fracture in patients with hypertension or obesity as well as in patients with diabetes for > 5 years. To the best of our knowledge, this is the first study to report the association of a 2-year weight loss or gain with the risk of fracture at various anatomical sites in patients with DKD, using annual health check-up data.

Previous general population studies have reported that a weight loss of ≥5% or 10% is associated with hip fracture in both postmenopausal women and men aged ≥ 50 (14, 19–22). Traditionally, obesity is considered to be protective against fractures owing to the long-term weight-bearing load effect that strengthens the bone. Therefore, low BMI, low weight, and significant weight loss are associated with increased fracture risk (23, 24). In recent studies that have focused on patients with diabetes, similar to the general population, weight loss was shown to increase the risk of frailty fracture (25, 26). Notably, the Action for Health in Diabetes trial showed that incident frailty fracture risk increased by ≥7% intentional weight loss among patients with type 2 diabetes and obesity (25). The Fukuoka Diabetes registry study illustrated that BW loss ≥ 20% from the maximum BW was associated with increased risk of hip and vertebral fractures in patients with type 2 diabetes (26), albeit the weight loss was not significantly associated with future fracture risk in women in subgroup analyses. This study, which included patients with DKD, showed that weight loss correlated with both vertebral and hip fractures, which is in line with previous results. In our subgroup analysis, the association between weight loss and fracture risk appeared to be stronger in men than in women with a significant interaction, consistent with results of the Fukuoka Diabetes registry study (26). The incidence rate of any-fracture was higher in women than in men in this study. Being a woman might have attenuated the relative risk of developing fractures due to weight loss. Therefore, weight loss may have exerted a relatively stronger effect on the development of any-fracture in men with DKD than in women.

Next, we evaluated weight gain as an independent risk factor for fracture in patients with DKD, as their association was unclear in this population. The relation between weight gain and fracture risk has been reported predominantly in postmenopausal women or older men in the general population, although findings to date have been inconsistent (14, 22). For example, a previous meta-analysis showed that weight gain may contribute to increased BMD and decreased hip fracture risk (22). In contrast, a > 5% weight gain in the Women's Health Initiative Observational study was not significantly associated with an increased risk of hip fracture but was associated with an increased incidence of upper and lower limb fractures in postmenopausal women (14). Indeed, previous studies have demonstrated a site-specific non-linear relationship between being overweight or having a BMI of 25–30 kg/m^2^ and fracture risk in the general population (27–29). In our study, DKD patients with ≥10% weight gain showed an increased risk for the development of any-fracture, including the spine and hip. Moreover, the adjusted HRs for any-fracture according to weight gain were higher in patients with obesity than in patients without obesity. Both diabetes and CKD have direct deleterious effects on qualitative bone microarchitecture and thus, contribute to fracture risk (30, 31). In this circumstance, weight gain might have aggravated the impaired mobility and increased traumatic forces, especially in DKD patients with obesity.

This study has some limitations. First, we did not have information regarding the BMD, and thus, could not determine the risk of osteoporotic fracture with changes in weight. Although low BMD predicts fracture risk in patients with CKD, non-osteoporotic causes of adynamic bone disease with low BMD could cause fractures in patients with diabetes and advanced CKD (32, 33). Second, we could not obtain information about intentional weight changes by the participants in this study. However, as previously mentioned, intentional weight loss was not associated with an increased risk of total or hip fractures in patients with diabetes (25). Third, the mean follow-up period in our study was shorter than that of previous studies, which have reported a mean fracture follow-up duration of approximately 11 years from baseline (14, 25). Forth, this data is retrospective and limited to Korean population. Despite these limitations, this study also has some strengths. To the best of our knowledge, this is the first study to examine the relationship between weight change and the incidence of vertebral, hip, or other fracture in a large sample size of 78,922 patients with DKD.

In conclusion, compared to patients with stable weight change, the risk of fracture at any site was increased in DKD patients who had a weight loss or gain ≥10%. Fracture risk was higher in men than in women, especially when the BW was lost. Furthermore, weight gain in patients with obesity was associated with an increased fracture risk. Therefore, patients with DKD who experience weight changes should be aware of the risk of fracture. Future prospective studies are needed to clarify the underlying mechanism by which weight changes increase the risk of fractures in patients with DKD.

## Data availability statement

Anonymized data are publicly available from the National Health Insurance Sharing Service and can be accessed at https://nhiss.nhis.or.kr/bd/ab/bdaba000eng.do.

## Ethics statement

This study was approved by the institutional review board of Chonnam National University Hospital, Korea (CNUH-EXP-2021-321) and performed in accordance with the ethical standards of the committee responsible for human experimentation and the Helsinki Declaration of 1975, as revised in 2013. The Ethics Committee waived the requirement of written informed consent for participation.

## Author contributions

CSK, KH, and SWK conceived and designed the study, and were responsible for the acquisition, analysis, and interpretation of the data. CSK, BK, and KH contributed to the statistical analysis. CSK and SWK contributed to the acquisition of funding for the study and drafted the manuscript. HSC, EHB, SKM, and SWK supervised the study. All authors critically revised the manuscript and approved the final version of the manuscript.

## Funding

This work was supported by the National Research Foundation of Korea (NRF) funded by the Korea Government (MSIT) (NRF-2019R1A2C2086276), by Basic Science Research Program through the of NRF funded by the Ministry of Education (NRF-2018R1D1A1B07042999), and by grants (BCRI22080 and BCRI21023) from Chonnam National University Hospital Biomedical Research Institute.

## Conflict of interest

The authors declare that the research was conducted in the absence of any commercial or financial relationships that could be construed as a potential conflict of interest.

## Publisher's note

All claims expressed in this article are solely those of the authors and do not necessarily represent those of their affiliated organizations, or those of the publisher, the editors and the reviewers. Any product that may be evaluated in this article, or claim that may be made by its manufacturer, is not guaranteed or endorsed by the publisher.
