# Supplementary material for: Weight change and fracture risk in patients with diabetic kidney disease: A nationwide population-based study
Source: Front Med (Lausanne). 2022 Jul 28;9:912152. doi: 10.3389/fmed.2022.912152 (PMC9366468; doi:10.3389/fmed.2022.912152)
Supplement: Supplementary file 1 [file Table_1.docx]

Supplementary Material

# Supplementary Tables

**Table S1. Sensitivity analysis of incidence rates and hazard ratios of fractures according to the status of weight changes over 4-years between the health check-ups.**

| Weight change | Number | Fracture | Follow-up Duration, Person-years | Incidence Rate, Per 1,000 person-years | Model 1, HR (95% CI) ^b^ | Model 2, HR (95% CI) ^c^ | Model 3, HR (95% CI) ^d^ |
| --- | --- | --- | --- | --- | --- | --- | --- |
| Any-fracture ^a^ |  |  |  |  |  |  |  |
| ≥ –10% | 3,971 | 467 | 14,202.39 | 32.88 | 1.248 (1.130, 1.378) | 1.238 (1.121, 1.367) | 1.253 (1.134, 1.384) |
| –10% to –5% | 9,322 | 894 | 35,295.95 | 25.33 | 1.073 (0.995, 1.158) | 1.073 (0.995, 1.158) | 1.081 (1.002, 1.166) |
| –5% to 5% | 32,909 | 2,683 | 126,319.19 | 21.24 | 1 (Reference) | 1 (Reference) | 1 (Reference) |
| 5% to 10% | 4,691 | 380 | 17,473.22 | 21.75 | 1.002 (0.900, 1.116) | 0.971 (0.872, 1.082) | 0.958 (0.860, 1.067) |
| ≥ 10% | 2,011 | 208 | 7,196.11 | 28.90 | 1.234 (1.071, 1.421) | 1.158 (1.005, 1.335) | 1.123 (0.973, 1.295) |
| Vertebral facture |  |  |  |  |  |  |  |
| ≥ –10% | 3,971 | 167 | 14,823.75 | 11.27 | 1.306 (1.105, 1.544) | 1.294 (1.094, 1.530) | 1.299 (1.098, 1.537) |
| –10% to –5% | 9,322 | 289 | 36,534.29 | 7.91 | 1.065 (0.932, 1.218) | 1.062 (0.929, 1.215) | 1.065 (0.931, 1.218) |
| –5% to 5% | 32,909 | 841 | 130,141.97 | 6.46 | 1 (Reference) | 1 (Reference) | 1 (Reference) |
| 5% to 10% | 4,691 | 118 | 18,007.25 | 6.55 | 0.996 (0.821, 1.208) | 0.985 (0.812, 1.195) | 0.98 (0.808, 1.190) |
| ≥ 10% | 2,011 | 74 | 7,446.52 | 9.9 | 1.363 (1.074, 1.729) | 1.330 (1.047, 1.688) | 1.315 (1.033, 1.673) |
| Hip fracture |  |  |  |  |  |  |  |
| ≥ –10% | 3,971 | 132 | 14,935.27 | 8.84 | 1.880 (1.545, 2.287) | 1.829 (1.503, 2.226) | 1.870 (1.536, 2.277) |
| –10% to –5% | 9,322 | 177 | 36,866.72 | 4.80 | 1.193 (1.002, 1.420) | 1.192 (1.001, 1.419) | 1.209 (1.016, 1.439) |
| –5% to 5% | 32,909 | 454 | 131,184.92 | 3.46 | 1 (Reference) | 1 (Reference) | 1 (Reference) |
| 5% to 10% | 4,691 | 59 | 18,132.54 | 3.25 | 0.944 (0.720, 1.238) | 0.885 (0.675, 1.162) | 0.857 (0.653, 1.126) |
| ≥ 10% | 2,011 | 41 | 7,527.01 | 5.45 | 1.430 (1.038, 1.969) | 1.265 (0.918, 1.744) | 1.177 (0.851, 1.627) |
| Other fractures |  |  |  |  |  |  |  |
| ≥ –10% | 3,971 | 225 | 14,704.19 | 15.30 | 1.064 (0.924, 1.224) | 1.065 (0.925, 1.226) | 1.078 (0.936, 1.241) |
| –10% to –5% | 9,322 | 496 | 36,073.91 | 13.75 | 1.039 (0.939, 1.15) | 1.045 (0.944, 1.156) | 1.052 (0.950, 1.164) |
| –5% to 5% | 32,909 | 1,575 | 128,511.76 | 12.26 | 1 (Reference) | 1 (Reference) | 1 (Reference) |
| 5% to 10% | 4,691 | 234 | 17,753.05 | 13.18 | 1.045 (0.911, 1.199) | 1.010 (0.880, 1.159) | 0.998 (0.869, 1.146) |
| ≥ 10% | 2,011 | 108 | 7,380.42 | 14.63 | 1.086 (0.894, 1.320) | 1.014 (0.834, 1.234) | 0.987 (0.810, 1.203) |

^a^ Composite fracture of the vertebral, hip, or other sites was defined as any-fracture.

^b^ Model 1, adjusted for age and sex.

^c^ Model 2, adjusted for age, sex, smoking, alcohol consumption, regular exercise, income status, use of insulin, more than 3 classes of oral hypoglycemic agents, diabetes duration, and previous histories of hypertension and dyslipidemia.

^d^ Model 3, adjusted for age, sex, smoking, alcohol consumption, regular exercise, income status, use of insulin, more than 3 classes of oral hypoglycemic agents, diabetes duration, previous histories of hypertension and dyslipidemia, and previous body weight.

Abbreviations: BP, blood pressure; CI, confidential interval; ESRD, end-stage renal disease; HR, hazard ratio.
